# The Association between Gestational Diabetes Mellitus and Infections in Pregnancy—Systematic Review and Meta-Analysis

**DOI:** 10.3390/microorganisms11081956

**Published:** 2023-07-31

**Authors:** Enav Yefet, Aviv Bejerano, Rula Iskander, Tal Zilberman Kimhi, Zohar Nachum

**Affiliations:** 1 Department of Obstetrics and Gynecology, Tzafon Medical Center, Poriya 1528001, Israel; 2 Azrieli Faculty of Medicine, Bar-Ilan University, Safed 1311502, Israel; 3 Department of Obstetrics and Gynecology, Emek Medical Center, Afula 1834111, Israelnachum.zo@gmail.com (Z.N.); 4 Rappaport Faculty of Medicine, Technion, Haifa 3109601, Israel

**Keywords:** gestational diabetes mellitus, vaginal infections, urinary tract infection (UTI), chorioamnionitis

## Abstract

We conducted a systematic review and meta-analysis to evaluate the association between gestational diabetes mellitus and infections during pregnancy. We included cross-sectional, case-control, cohort studies and clinical trials, evaluating the frequency of infections in women with and without gestational diabetes mellitus. A search was conducted in Embase, PubMed, and Web of Science electronic databases and by manually searching references, until 23 March 2022, resulting in 16 studies being selected for review, with 111,649 women in the gestational diabetes mellitus group, and 1,429,659 in the controls. Cochrane’s Q test of heterogeneity and I² were used to assess heterogeneity. Pooled odds ratio (OR) was calculated. Funnel plots and Egger test were used for assessment of publication bias. The results showed a significant association between gestational diabetes mellitus and infections (pooled-OR 1.3 95% CI [1.2–1.5]). Sub-analyses showed a significant association for urinary tract infections (pooled-OR of 1.2 95% CI [1.1–1.3]), bacterial infections (pooled-OR were 1.2 95% CI [1.1–1.4]), and SARS-CoV-2 (pooled-OR 1.5 95% CI [1.2–2.0]) but not to gingivitis or vaginal candidiasis. The results underscore the significance of acknowledging gestational diabetes mellitus as a risk factor for infections.

## 1. Introduction

Gestational diabetes mellitus is the most common metabolic disorder of pregnancy. The prevalence of this disease is ranging from 0.6 to 15% and is dependent on race, ethnicity, location, season, and socio-economic status [1,2,3]. The diagnosis method of gestational diabetes mellitus also contributes to the different prevalence among different countries and different regions within the same country. The use of the international association of the diabetes and pregnancy study groups (IADPSG) criteria has led to increased incidences of gestational diabetes mellitus reaching 27.5% in Southern Italy and 41.9% in North Indian women [1,2,3,4].

In this condition that arises during pregnancy, pancreatic function is inadequate to overcome insulin resistance associated with the pregnant state. Gestational diabetes mellitus is characterized by hyperglycemia causing significant morbidity for both mother and child [5,6,7,8,9,10].

Adverse maternal outcomes include increased risk for cesarean delivery, pregnancy-induced hypertension, post-partum hemorrhage, severe perineal and anal sphincter lacerations, and an increased risk for future type 2 diabetes mellitus. Adverse perinatal outcomes include an increased risk for fetal macrosomia, a large gestational age fetus, an increased mean birth weight, neonatal hypoglycemia, and an Apgar score of less than 7 at 5 min after delivery [11,12,13,14]. Research indicates that gestational diabetes mellitus may be linked to an increased incidence of infections, such as vaginal infections, urinary tract infections, and chorioamnionitis [9,15,16].

Due to its association to a poor metabolic control, higher body mass index, impaired leukocyte function, and a change in vaginal pH [9,17,18,19,20], some studies suggest that gestational diabetes mellitus is linked to disturbances in the vaginal flora and vaginal infections [21,22,23,24,25]. 

Infections during pregnancy are closely associated with adverse pregnancy outcomes, such as premature rupture of membranes, puerperal infection, preterm delivery, intrauterine infections, stillbirth, and neurological damage to the fetus [9,26,27]. Consequently, a better understanding of the interconnection between gestational diabetes mellitus and infections, including mechanisms and possible outcomes, could potentially lead to better and more accurate recommendations, screening tests, and treatment regimens, which can ultimately aid in reducing the morbidity among women with gestational diabetes mellitus and their unborn fetuses.

Nevertheless, opinions on the subject are divided [28,29,30], and the association between gestational diabetes mellitus and infections during pregnancy remains unclear. Accurate knowledge of this association is essential as it can aid in developing better screening tests and reducing morbidity.

We aimed to conduct a systematic review and meta-analysis to evaluate the association between gestational diabetes mellitus and infections during pregnancy.

## 2. Materials and Methods

### 2.1. Eligibility Criteria, Information Sources, and Search Strategy

The present study employed a systematic review and meta-analysis, which were registered in PROSPERO (international prospective register for systematic reviews, University of York, York, UK) under the assigned registration number (CRD42022359408). This meta-analysis was performed according to the guidelines for the systematic review and meta-analysis of observational studies in epidemiology (MOOSE) checklist [31]. Embase, PubMed, Ovid-Medline, and Web of Science were searched using the following keywords: gestational diabetes, diabetes mellitus gravidarum, pregnancy diabetes, gestational diabetes mellitus, and infection/s. The inclusion criteria for this study consisted of epidemiological studies, including cross-sectional, case-control, cohort studies and clinical trials, evaluating the frequency of infections in women with gestational diabetes mellitus. These studies were required to include a control group of healthy pregnant women without gestational diabetes mellitus. For the initial search, there was no limitation with respect to the gestational diabetes mellitus diagnosis criteria, the type of infection, or the diagnosis method. Studies excluded from our analysis included systematic reviews, literature reviews, comments to the editor, studies published in the form of conference proceedings, studies without a control group, and studies published in a language other than English. A search was conducted in 23 March 2022. In addition, the reference lists were searched manually for additional manuscripts, including reviews. In cases where there were incomplete data, or the full texts were unavailable, attempts to contact the authors were made and interlibrary loans were used.

Two independent authors with training in medical database searching (AB and RI) screened independently the titles and abstracts of the manuscripts for eligibility, and later on, the full manuscripts were reviewed for appropriateness. The percent of agreement between the authors was calculated as well as the unweighted kappa value (κ). K > 0.4, suggesting at least moderate agreement, was considered acceptable. Disagreements were resolved by the study’s moderators (EY and ZN).

### 2.2. Data Extraction

Data extraction was performed by one author (AB) and reviewed for accuracy by the study’s moderators (EY and ZN). The following were extracted: first author name, publication year, location of the study, study design, whether it was a single center or multicenter study, gestational diabetes mellitus diagnosis criteria, and number of patients in each study group.

### 2.3. Outcomes

The primary outcome was the rate of infections during pregnancy. Secondary outcomes were the rate of individual infections, such as bacterial vaginosis, vaginal mycosis, urinary tract infections, and gingivitis. A pooled odds ratio (OR) with 95% confidence interval (CI) was calculated for the study outcomes. We performed sub-analyses, in which we examined the association between gestational diabetes mellitus, type of pathogen (bacterial, viral, and mycosis), and gestational diabetes mellitus diagnostic criteria.

### 2.4. Data Synthesis and Assessment of the Risk of Bias

All reports were assigned a quality score based on the strengthening the reporting of observational studies in epidemiology (STROBE) checklist [32]. Quality assessment was performed using the Newcastle-Ottawa Scale for cohort and case-control studies [33]. A total score lower than 7 stars out of 9 was considered as an elevated risk of bias. 

The quality of the body of evidence for the outcome (infections) was assessed according to the grading of recommendations assessment, development, and evaluation (GRADE) system [34]. We assessed the body of evidence based on inconsistency, risk of bias, imprecision, indirectness, and publication bias. The certainty of the evidence was reflected by the overall rating. 

Cochrane’s Q test of heterogeneity was used to assess the heterogeneity of the studies. Inconsistencies in study results were assessed by I². We used the random effects model (DerSimonian and Laird) if the Cochrane’s Q test was *p* < 0.1 or I² ≥ 50%. Otherwise, we used the fixed effects model (inverse variance methods). The funnel plot and the Egger test were used to assess publication bias (*p* < 0.05 was defined as a statistically asymmetric funnel plot). Meta-analyses and review articles are exempt from the institutional review board approval in our institutions.

Trial registration: This study was registered at the international prospective register of systematic reviews (PROSPERO) (CRD42022359408).

## 3. Results

### 3.1. General Features

**Figure 1:**
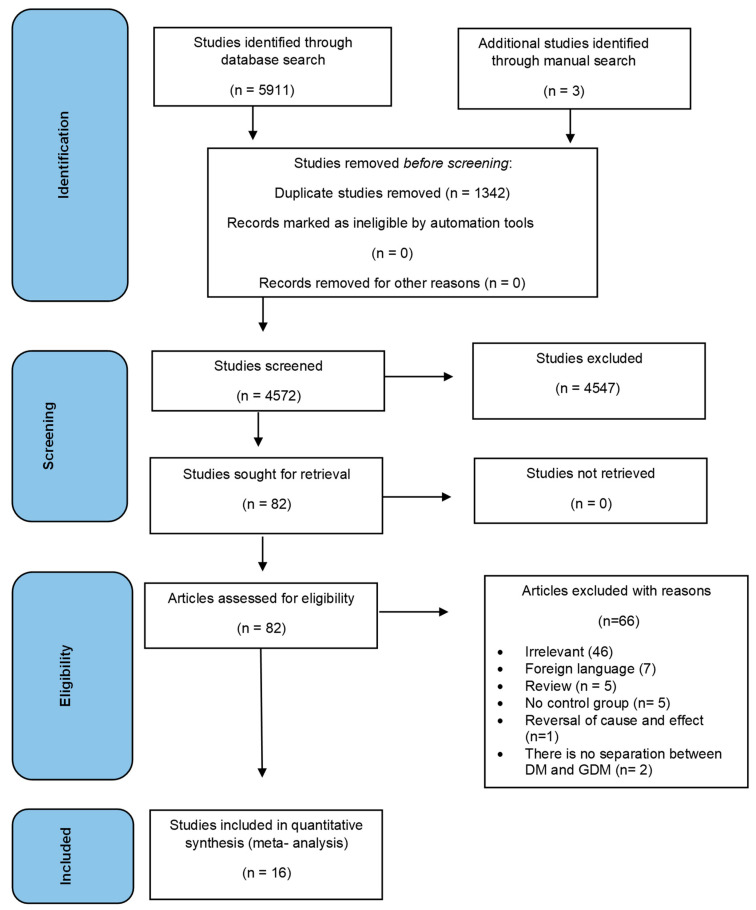
Study selection process.

During the initial database search, a total of 5911 citations were identified based on the predefined exclusion and inclusion criteria. Three additional studies were identified through manual search. After removing 1342 duplicate articles, a total of 4572 articles were selected for further analysis. The study selection process is described in Figure 1. Finally, 16 studies published between 1999 and 2022 were selected for systematic review. The percent of agreement between the authors was 99% and the unweighted kappa value (κ) was 0.46, suggesting moderate agreement. 

Table 1 displays the study characteristics, encompassing information on studies conducted in various countries. Of the studies conducted, seven of them were multicenter. The aggregate number of women included in these studies amounted to 1,575,822, including 111,649 women in the gestational diabetes mellitus group and 1,429,659 in the control group of pregnant women without gestational diabetes mellitus. Notably, there was a significant heterogeneity observed in the pathogens that caused the infection. The quality of the included studies as evaluated by the Newcastle-Ottawa Scale is presented in Table 2. Fifteen out of 16 studies scored between 7 and 9 of 9 stars, indicating high quality and a low risk of bias. The remaining study scored 6 of 9 stars, indicating an elevated risk of bias. 

### 3.2. Association between Gestational Diabetes Mellitus and Infections

**Figure 2:**
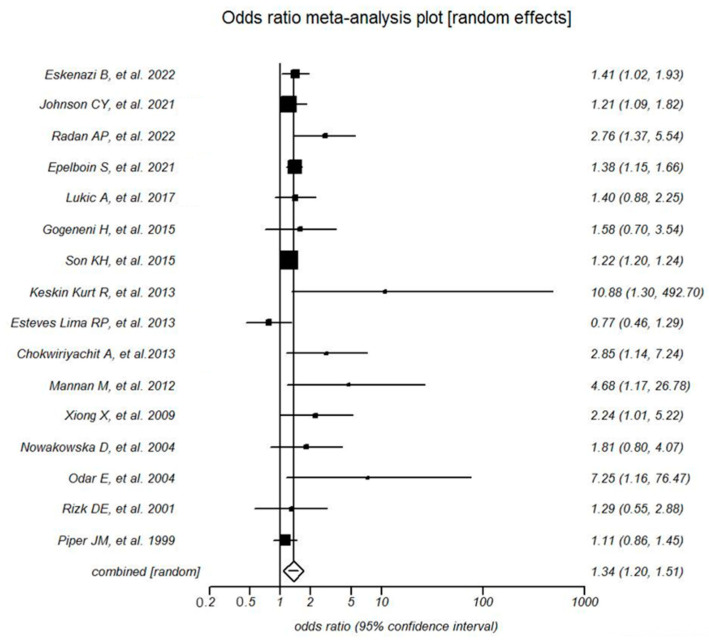
Forest plot representing the association between gestational diabetes mellitus and infections in various studies [15,24,28,35,36,37,38,39,40,41,42,43,44,45,46,47].

**Figure 3:**
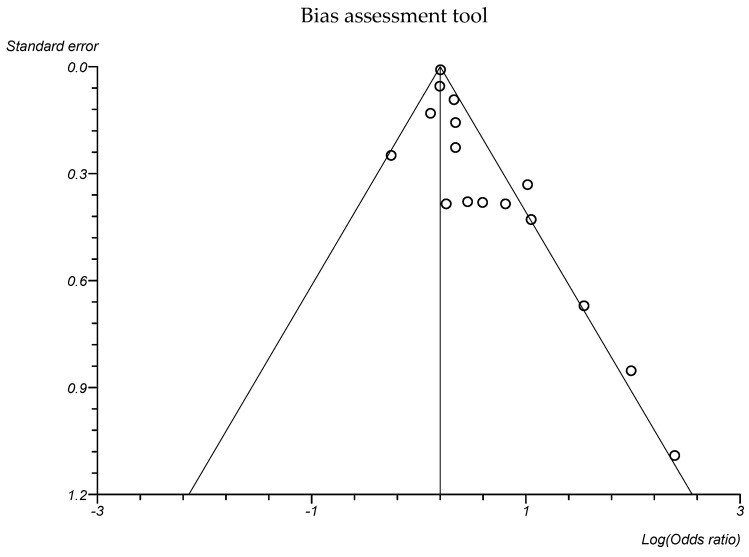
Funnel plot for studies evaluating the association between gestational diabetes mellitus and infections.

The statistical analysis of the data is presented in the forest plot at Figure 2. A random effects model was used to estimate the pooled OR and its 95% CI. The risk for infections during pregnancy was higher in women with gestational diabetes mellitus compared with women without gestational diabetes mellitus (pooled OR 1.3 a 95% CI 1.2 to 1.5, *p* < 0.0001; Grade: low). Publication bias was suggested by the Egger’s test (*p* = 0.007) and the funnel plot (Figure 3).

### 3.3. Association between Gestational Diabetes Mellitus and Type of Infection Site

We performed a sub-analysis for urinary tract infections and gingivitis, the most studied infections sites. For each type of infection site, a random effects model was used.

For urinary tract infections, the meta-analysis showed a significant association with gestational diabetes mellitus, with a pooled OR of 1.2 95% CI 1.1 to 1.3. The Egger’s test showed no significant publication bias (*p* = 0.39).

For gingivitis, the meta-analysis showed a non-significant association with gestational diabetes mellitus, with a pooled OR of 1.6 95% CI 0.8 to 3.0. The Egger’s test showed a significant publication bias (*p* = 0.02).

### 3.4. Association between Gestational Diabetes Mellitus and Type of Pathogens

To investigate the association between gestational diabetes mellitus and different types of pathogens, we conducted a sub-analysis of three types of infections: bacterial infections, mycosis infections, and SARS-CoV-2 infections. We used a random effects model to all analyses.

For bacterial infections and SARS-CoV-2, the pooled ORs were 1.2 95% CI 1.1 to 1.4 and 1.5 95% CI 1.2 to 2.0, respectively, indicating a significant positive association with gestational diabetes mellitus. Mycosis infections were not associated with gestational diabetes mellitus (pooled OR 2.9 95% CI 0.8 to 10.8).

### 3.5. Association between Gestational Diabetes Mellitus and Diagnostic Criteria 

Different diagnostic criteria were used in different studies. Three studies used the 75 g oral glucose tolerance test criteria of the IADPSG (Table 1), which were adopted by the American Diabetes Association [48]. In a sub-analysis of the association between gestational diabetes mellitus and infections in those studies, there was no significant difference in the rate of infections between women with (224 women) and without (477 women) gestational diabetes mellitus (pooled OR 1.46 with 95% CI 0.66 to 3.24, random effects model).

Four studies used the 100 g oral glucose tolerance test criteria of either Carpenter and Coustan criteria or the National Diabetes Data Group (Table 1), which are accepted by the American College of Obstetricians and Gynecologists [11]. In a sub-analysis of the association between gestational diabetes mellitus and infections in those studies, the gestational diabetes mellitus group had a significantly higher rate of infections (582 women) compared with the control group (1232 women) (pooled OR 2.10 with 95% CI 1.03 to 4.29, random effect model).

## 4. Discussion

Our aim was to systematically review all studies on the association between gestational diabetes mellitus and infections. Our meta-analysis results demonstrated a significant association between gestational diabetes mellitus and infections. Sub-analyses showed a significant association for urinary tract infections, bacterial infections, and SARS-CoV-2 infections but not to gingivitis or vaginal mycosis. 

Diabetes mellitus is a known risk factor for infections. In a meta-analysis of 345 observational studies, the association between diabetes mellitus and the risk of incident infections in adults was quantified. Diabetes mellitus increased significantly the risk of infection of the skin (Odds ratio (OR) 1.94, 95% CI 1.78 to 2.12), respiratory (OR 1.35, 95% CI 1.28 to 1.43), blood (OR 1.72, 95% CI 1.48 to 2.00), genitourinary (OR 1.61, 95% CI 1.42 to 1.82), head and neck (OR 1.17, 95% CI 1.13 to 1.22), gastrointestinal (OR 1.48, 95% CI 1.40 to 1.57), viral (OR 1.29, 95% CI 1.13 to 1.46), and non-specified infections (OR 1.84, 95% CI 1.66 to 2.04) [49].

In a meta-analysis that evaluated whether asymptomatic bacteriuria is more common in patients with diabetes mellitus than among control subjects, it was found that asymptomatic bacteriuria was present in 439 of 3579 (12%) patients with diabetes mellitus and in 121 of 2702 (4.5%) healthy control subjects. Asymptomatic bacteriuria was more common both in patients with type 1 diabetes mellitus (OR 3.0) and type 2 diabetes mellitus (OR 3.2) than in control subjects [40]. It was hypothesized that immune system dysfunction in patients with diabetes mellitus could be mediated through impaired migration, phagocytosis, intracellular killing, and chemotaxis of the immune cells [50,51,52]. Previous studies indicated that hormonal level changes and maternal body habitat microbiome alterations during pregnancy can affect the immune response and increase the risk of infections [9,20,53,54,55]. One possible explanation for the increased risk for infections in gestational diabetes mellitus is that gestational diabetes mellitus may compromise the immune system making women more susceptible to infections [17,18,56]. Hyperglycemia associated with gestational diabetes mellitus can impair neutrophil function, leading to a decreased ability to fight off infections [57]. In the study of Koren et al. [51], it was shown that both healthy pregnant women and those with gestational diabetes mellitus displayed changes in the composition of their gut microbiota with advancing gestational age. However, the women who later developed gestational diabetes mellitus had lower biodiversity of the intestinal microbiota in the first trimester. It was also shown that women with higher insulin resistance, higher glycemic levels, and adiposity had increased markers of inflammation in stool samples collected from the first and third trimesters of pregnancy.

Studies also suggested that there was a direct association between poor glycemic control and an increased incidence of infections [9,18,39]. It was shown that suboptimal glycemic control could affect the composition of the vaginal microbiome [58]. A healthy vaginal microbiome plays a crucial role in preventing bacterial vaginosis, vaginal candidiasis, and other bacterial infections [59,60]. The vaginal microbiome comprises beneficial microorganisms that independently perform specific functions to preserve the healthy state of the vaginal tract. As previously mentioned, lactobacillus accounts for around 70% of the vaginal flora [9,61]. Lactobacillus is believed to exert a protective role by neutralizing the deleterious impact of pathogenic microbes through the generation of an acidic environment via lactic acid production [60]. Specifically, gestational diabetes mellitus was shown to shift the vaginal microbiome from lactobacilli crispatus to lactobacilli acidophilus colonization [9]. The extent to which this may causally alter infection rate is yet to be determined in future studies. 

Identifying gestational diabetes mellitus as a risk factor for maternal asymptomatic bacteriuria is important since it was associated with infectious disease of the offspring later in life. In a study that aimed to test the association between maternal asymptomatic bacteriuria during pregnancy and long term offspring infectious hospitalizations, it was found that asymptomatic bacteriuria during pregnancy increased offspring susceptibility to long-term infectious hospitalizations even after controlling for maternal age, diabetes mellitus, ethnicity, hypertensive disorders, and gestational age, thus, emphasizing the importance of screening high-risk populations for maternal asymptomatic bacteriuria, including women with gestational diabetes mellitus [62].

The hypothesis regarding urinary tract infections and pregnancy is that anatomical and physiological changes during pregnancy facilitate bacterial growth and the ascent of bacteria toward the kidneys. It was previously thought that gestational diabetes mellitus could be an additional risk factor for asymptomatic bacteriuria and urinary tract infections [63,64]. Evidence suggests that pregnant women with asymptomatic bacteriuria are more likely to develop symptomatic urinary tract infections when they have gestational diabetes mellitus, compared to those without gestational diabetes mellitus [65]. Glycosuria, which is more common in gestational diabetes mellitus, encourages bacterial growth in the urine. Combining with physiological changes in pregnancy, such as ureteral dilatation, increased bladder volume, and decreased bladder tone, along with decreased ureteral tone, and an increase in urinary estrogens and progestins, may lead to a reduced ability of the lower urinary tract to oppose invading bacteria. In women with gestational diabetes mellitus, these factors can all contribute to the development of urinary tract infections [66].

An alternative hypothesis for the observed outcomes is the reversal of causation, where infections could potentially play a role in the onset of gestational diabetes mellitus. [67] Infections can cause inflammation and oxidative stress, both of which can lead to insulin resistance and impaired glucose metabolism [68,69,70]. Furthermore, some pathogens, such as viruses, may directly infect pancreatic beta cells, resulting in reduced insulin secretion and impaired glucose tolerance [71,72]. The exact mechanisms underlying the observed association between gestational diabetes mellitus and infection are still not fully understood, and further research is needed to elucidate these mechanisms. 

In this meta-analysis, we found a positive association between gestational diabetes mellitus and SARS-CoV-2 infection. When endothelial cells were cultured with an increasing concentration gradient of SARS-CoV-2 spike protein (S protein) within differing glucose mediums, it was demonstrated that a high glucose medium led to an aggravation of the decrease in angiotensin-converting enzyme 2 (ACE2) and activation of nicotinamide adenine dinucleotide phosphate (NADPH) oxidase (NOX) 2 and NOX4 in cultured cells. S protein-induced oxidative stress and apoptosis were mediated by activation of the ACE2-NOX axis within endothelial cells, causing a reduction in nitric oxide and tight junction proteins, leading to cellular dysfunction [73]. 

There were only two studies that evaluated the association between vaginal mycosis and gestational diabetes mellitus. Both of them were from 2004. In one study, the risk for vaginal mycosis was six times higher in women with gestational diabetes mellitus than in women without gestational diabetes mellitus [46]. In the second study, the risk for vaginal mycosis was twice as high as the risk in women without gestational diabetes mellitus, but this risk did not reach statistical significance [24]. Although the risk for vaginal mycosis in women with gestational diabetes mellitus was not statistically significant in this meta-analysis, the available data are very modest, and the trend toward positive association of vaginal mycosis with gestational diabetes mellitus merits further exploration.

In a sub-analysis, gestational diabetes mellitus, according to 100 g oral glucose tolerance test criteria but not the 75 g IADPSG criteria, was associated with a higher rate of infections. Possible explanations are the smaller sample size of the studies, which used the IADPSG criteria, and the fact that women with milder hyperglycemia are considered as having gestational diabetes mellitus, which might weaken the effect on the rate of infections.

The findings of this meta-analysis have important implications for clinical practice. The increased risk for urinary tract infections may encourage more intense screening and treatment of asymptomatic bacteriuria. Moreover, women with gestational diabetes mellitus should be informed regarding the increased risk for SARS-CoV-2 infection, and vaccination should be offered. Future studies should evaluate the importance of monitoring and managing other infections in pregnant women with gestational diabetes mellitus to reduce adverse outcomes for both the mother and the child. Finally, further research is necessary to determine the underlying mechanisms and develop effective strategies for prevention and management of infections in this population.

Our study’s strengths are its incorporation of several high-quality studies with an average STROBE score of 18.5, including multi-center studies with numerous participants. Ultimately, we narrowed our focus to 16 articles that were included in our final meta-analysis. Enough studies allowed us to conduct multiple sub-analyses, allowing us to carefully examine the causes of heterogeneity and distinguish between different types of infections that may have varying associations with gestational diabetes mellitus.

Nonetheless, there are several potential limitations that must be taken into account. The heterogeneity across the studies included in this meta-analysis is a crucial factor to consider while interpreting the results. The studies varied in their design, sample size, and gestational diabetes mellitus diagnostic method, which could have contributed to the observed heterogeneity. Additionally, the definition of infectious disease differed among the studies, particularly those that examined the association between gestational diabetes mellitus and gingivitis. Another crucial limitation to acknowledge is the possibility of publication bias, as indicated by the Egger’s test performed in this research. Negative studies may be less likely to be published, resulting in an overestimation of the true effect size. Therefore, further investigation is necessary to validate these findings and to address potential sources of bias.

## 5. Conclusions

This systematic review and meta-analysis of 16 studies involving over 1.5 million women with gestational diabetes mellitus and controls of healthy pregnant women without gestational diabetes mellitus demonstrated a significant association between gestational diabetes mellitus and infections, particularly urinary tract infections, bacterial infections, and SARS-CoV-2 infection. The results underscore the significance of acknowledging gestational diabetes mellitus as a risk factor for infections. 

## Figures and Tables

**Table 1:** Characteristics of Included Studies.

Authors	Location of Study	Study Design	Multicenter	Type of Infection	GDM Diagnosis Criteria	Number of Total Patients	Number of Patients without GDM with Infection/Total without GDM	Number of Patients with GDM with Infection/Total GDM	Strobe Score (Out of 22)
Eskenazi B, et al. 2022 [35]	Multinational 18 countries	Prospective cohort study	Yes	SARS-CoV-2	Abstracted from medical records	2071	564/1824	75/194	20
Johnson CY, et al. 2021 [36]	USA	Case control study	Yes	urinary tract infections	N/A	41,869	7003/38,908	445/2118	18
Radan AP, et al. 2022 [37]	Switzerland	Case control study	No	SARS-CoV-2	IADPSG criteria	224	24/149	26/75	20
Epelboin S, et al. 2021 [38]	France	Retrospective cohort study	Yes	SARS-CoV-2	ICD 10 codes	244,645	735/214,735	139/29,251	22
Lukic A, et al. 2017 [39]	Italy	Prospective cohort study	Yes	Cervicovaginal Bacteria—group B Streptococcus, *Gardnerella vaginalis*, *Candida* spp., *Chlamydia trachomatis*, *Mycoplasma hominis*, and *Ureaplasma urealyticum*	N/A	473	169/346	59/103	19
Gogeneni H, et al. 2015 [40]	Turkey	Case control study	No	Gingivitis—*Porphyromonas gingivalis*, *Filifactor alocis* and *Treponema denticola*	IADPSG criteria	117	31/58	38/59	18
Son KH, et al. 2015 [41]	South Korea	Retrospective cohort study	Yes	Infection of genitourinary tract	ICD-10 codes (Data from National Health Insurance)	1,282,498	246,378/1,171,575	19,323/78,716	18
Keskin Kurt R, et al. 2013 [42]	Turkey	Prospective cohort study	No	*Demodex folliculorum*	Carpenter and Coustan criteria	66	30-Jan	9/33	18
Esteves Lima RP, et al. 2013 [43]	Brazil	Case control study	No	Periodontitis	IADPSG criteria	360	125/270	36/90	20
Chokwiriyachit A, et al.2013 [44]	Thailand	Case control study	Yes	Periodontitis	the NDDG criteria	100	13/50	25/50	18
Mannan M, et al. 2012 [15]	Bangladesh	Cross-sectional study	Yes	urinary tract infections	Modified method of Carpenter and Coustan criteria following 75 g OGTT	960	3/72	12/71	19
Xiong X, et al. 2009 [45]	USA	Case control study	No	Periodontitis	Carpenter and Coustan criteria	159	64/102	41/53	20
Odar E, et al. 2004 [46]	Uganda	Prospective cohort study	No	*Vaginal candidiasis*	The WHO criteria for diagnosis of diabetes—two-hour 75 g oral glucose load	90	2/60	30-Jun	19
Nowakowska D, et al. 2004 [24]	Poland	Cross-sectional study	No	*Vaginal mycosis*	N/A	251	18/132	16/72	14
Rizk DE, et al. 2001 [28]	United Arab Emirates	Prospective cohort study	No	*E. coli*, *Klebsiella pneumonia*, *Proteus mirabilis*, *Staphylococcus aureus*, and group B streptococcus *Asymptomatic bacteriuria* Symptomatic urinary tract infections *Acute cystitis* *Acute pyelonephritis*	Venous plasma glucose levels of 5.3 mmol/L after fasting and/or 8.6 mmol/L 2 h after an oral 75 g glucose load	447	19/298	12/149	16
Piper JM, et al. 1999 [47]	USA	Prospective cohort study	No	Group B Streptococcus colonization	At least one abnormal OGTT value according to the NDDG criteria	1492	253/1046	117/466	18

IADPSG, international association of diabetes and pregnancy study groups; NDDG, national diabetes data group; OGTT, oral glucose tolerance test.

**Table 2:** Newcastle-Ottawa Scale for quality assessment of cohort and case-control studies.

Authors	Selection				Comparability of Groups	Outcome/ Exposure			Total	Study Design
	1	2	3	4	5	6	7	8		
Eskenazi B, et al. 2022 [35]	*	*	*	*	**	*	*	*	9	Prospective cohort study
Johnson CY, et al. 2021 [36]			*	*	**	*	*	*	7	Case control study
Radan AP, et al. 2022 [37]	*	*		*	**	*	*	*	8	Case control study
Epelboin S, et al. 2021 [38]	*	*	*		**	*	*	*	8	Retrospective cohort study
Lukic A, et al. 2017 [39]	*	*		*	**	*		*	7	Prospective cohort study
Gogeneni H, et al. 2015 [40]	*	*	*	*		*	*	*	7	Case control study
Son KH, et al. 2015 [41]	*	*	*			*	*	*	6	Retrospective cohort study
Keskin Kurt R, et al. 2013 [42]	*	*	*	*	**	*	*	*	9	Prospective cohort study
Esteves Lima RP, et al. 2013 [43]	*		*	*	**	*	*	*	8	Case control study
Chokwiriyachit A, et al.2013 [44]	*	*	*	*	**	*	*	*	9	Case control study
Mannan M, et al. 2012 [15]	*	*	*		**	*	*	*	8	Cross-sectional study
Xiong X, et al. 2009 [45]	*	*	*	*	**	*	*	*	9	Case control study
Odar E, et al. 2004 [46]	*	*	*	*		*	*	*	7	Prospective cohort study
Nowakowska D, et al. 2004 [24]	*	*	*		**	*	*	*	8	Cross-sectional study
Rizk DE, et al. 2001 [28]	*	*	*	*	**	*	*	*	9	Prospective cohort study
Piper JM, et al. 1999 [47]	*	*	*		**	*	*	*	8	Prospective cohort study

Each * represents one point in the Newcastle-Ottawa Scale.

## Data Availability

No new data were generated during this meta-analysis.
